# Stromal–immune cell crosstalk fundamentally alters the lung microenvironment following tissue insult

**DOI:** 10.1111/imm.13319

**Published:** 2021-03-11

**Authors:** Julie C. Worrell, Megan K. L. MacLeod

**Affiliations:** ^1^ Institute of Infection, Immunity and Inflammation University of Glasgow Glasgow UK

**Keywords:** epithelial cells, fibroblasts, lung, stromal–immune cell interactions

## Abstract

Communication between stromal and immune cells is essential to maintain tissue homeostasis, mount an effective immune response and promote tissue repair. This ‘crosstalk’ occurs in both the steady state and following a variety of insults, for example, in response to local injury, at sites of infection or cancer. What do we mean by crosstalk between cells? Reciprocal activation and/or regulation occurs between immune and stromal cells, by direct cell contact and indirect mechanisms, including the release of soluble cytokines. Moving beyond cell‐to‐cell contact, this review investigates the complexity of ‘cross‐space’ cellular communication. We highlight different examples of cellular communication by a variety of lung stromal and immune cells following tissue insults. This review examines how the ‘geography of the lung microenvironment’ is altered in various disease states; more specifically, we investigate how this influences lung epithelial cells and fibroblasts *via* their communication with immune cells and each other.

## Graphical abstract

Lung stromal cells are altered in various disease states, this influences their communication with immune cells and each other. Stromal cells can perform functions classically attributed to immune cells, providing immunomodulatory functions and educating local immune cells within the lung. Communication between immune and stromal cells provides mutual support for the persistence of both cell types.

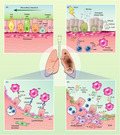

**Abbreviations**
ADI: alveolar intermediate differentiation
AM: alveolar macrophage
ATI: alveolar type I epithelial cell
ATII: alveolar type II epithelial cell
CAFs: cancer‐associated fibroblasts
CCL: C‐C motif chemokine ligand
CSF: colony‐stimulating factor
CXCL: C‐X‐C motif chemokine ligand
CXCR: C‐X‐C motif chemokine receptor
ECM: extracellular matrix
HEV: high endothelial venule
IAV: influenza virus
iBALT: inducible bronchus‐associated tissue
IFN: interferon
IL: interleukin
IPF: idiopathic pulmonary fibrosis
KRT5: keratin‐5
LCMV‐Cl13: lymphocytic choriomeningitis virus Clone 13
LEC: lymphatic endothelial cell
*M.tb*: *Mycobacterium tuberculosis*
MMP: matrix metalloproteinases
OSM: oncostatin M
PDGF: platelet‐derived growth factor
PDPN: podoplanin
ROS: radical oxygen species
SARS‐CoV‐2: severe acute respiratory syndrome coronavirus 2
SCC: solitary chemosensory cell
sc‐RNA‐seq: single‐cell RNA‐sequencing
TGF: transforming growth factor
TNF‐α: tumour necrosis factor‐α
VEGF: vascular endothelial growth factor
α‐SMA: alpha‐smooth muscle actin

## Introduction

The interactions between stromal and immune cells during the steady state and in response to disease causing agents are complex, encompassing a vast network of interactions and regulatory mechanisms.^1^, ^2^ Stromal cells, including epithelial, endothelial cells and fibroblasts, provide a structural framework that enables haematopoietic cells to carry out their functions. Stromal cells provide, however, much more structural support.^3^, ^4^ Immune cells (tissue‐resident and those carrying out surveillance around the body) are instructed by resident stromal cells in their development, survival and function. Interactions between stromal and immune cells must be tightly regulated, failure of cell communications can result in aberrant repair processes, uncontrolled cell growth and cancer.^5^

The respiratory epithelium is a ‘front line’ of defence and is exposed to a variety of atmospheric contaminants and noxious stimuli including pollution, cigarette smoke, respiratory microbes and allergens. Epithelial cells are susceptible to damage in a variety of lung injury models including the bleomycin model of pulmonary fibrosis, in partial pneumonectomy and following influenza virus infection.^6^, ^7^, ^8^ Pulmonary fibroblasts respond to environmental signals triggered by injury or infections, and the controlled accumulation of fibroblasts to sites of inflammation is crucial for effective tissue repair.^9^, ^10^ Classically, fibroblast activation occurs following lung injury leading to myofibroblast differentiation and expression of alpha‐smooth muscle actin (α‐SMA).^11^ Destruction and aberrant remodelling of extracellular matrix (ECM) is a common pathological feature of several diseases including pulmonary fibrosis,^12^ asthma,^13^ lung cancer^14^ and chronic viral infections.^10^

## New technologies can reveal receptor–ligand interactions and provide a spatial context to immune–stromal cell communication

Signals delivered by one cell to another *via* cell surface molecules require direct cell–cell contact. In protective immune responses, important interactions of this type include the killing of infected cells by CD8 T cells *via* Fas/FasL interactions.^15^, ^16^ Direct contact between stromal and immune cells is also required to maintain lung homeostasis. For example, inhibitory signals through the receptor–ligand interaction CD200‐CD200R on lung epithelial cells and alveolar macrophages require physical proximity and act to encourage a return to the resting state following infection.^17^

Paracrine cell–cell communication does not require direct cell–cell contact but depends on the diffusion of signalling molecules from one cell to another. Interactions between immune and stromal cells often occur following the release of soluble, cell‐derived cytokines and chemokines (e.g. interferon‐alpha (IFN‐α) and C‐X‐C motif chemokine ligand (CXCL10)). Non‐haematopoietic stromal cells express the cognate receptors for these molecules^18^ and following tissue injury are capable of inducing bidirectional activation of circulating immune cells *via* the production of chemokines, for example CXCL10.

Progress in transcriptomic analysis, including single‐cell RNA‐sequencing (scRNA‐seq), is increasing the depth of understanding of cellular crosstalk in homeostasis and in disease states. Spatial transcriptomics can provide this information in the context of the tissue microenvironment by monitoring gene expression in intact tissue sections rather than dissociated cells. Analysis of these data using tools such as CellPhoneDB^19^ (~ 900 receptor–ligand pairs) and CellTalker^20^ (~ 2000 receptor–ligand pairs) allows us to interrogate our knowledge and predict receptor–ligand interactions.

Expression of mRNA of receptor/ligand pairs in dissociated tissues is not sufficient to determine cell–cell interactions. The molecules must be localized in the correct cellular compartment as soluble molecules, such as cytokines, usually act locally.^21^ Visualization of interactions or close proximity between neighbouring cells can be examined using microscopy. Immunofluorescent and immunohistochemical methods can be combined for protein detection and *in situ* hybridization approaches, such as RNA‐scope, used to visualize gene expression. By adding further spatial context to ligand–receptor interactions, it is possible to attribute functional properties to cells based on anatomical location. A combination of these approaches may be ideal to investigate transcriptional profiles of all lung‐resident stromal cells and allow spatial allocation to distinct microenvironmental niches. For example, studies investigating how the lung response to injury alters cellular communication^22^, ^23^ identified a new subset of endothelial cells capable of communication with neighbouring alveolar epithelial cells through vascular endothelial growth factor (VEGF) signalling.^23^

Transcriptional studies have highlighted that fibroblasts display ‘positional identity’, with distinct transcriptomes depending on tissue and within‐tissue location.^24^, ^25^ Importantly, several recent studies in the human and mouse lung illustrate the switch between stromal–stromal and stromal–immune cell interactions in disease states.^25^, ^26^, ^27^, ^28^, ^29^ Multiple studies also highlight the biological relevance of profiling of different cell types in adult tissues, demonstrating how altered cell–cell communication following infection or disease results in specific ‘networks’ across space and local microenvironmental niches.^22^, ^23^, ^26^, ^30^, ^31^

## Pulmonary homeostasis and stromal cell heterogeneity

The lung consists of a diverse population of stromal cells that act in concert with innate and adaptive immune cells to maintain and restore pulmonary homeostasis. The upper respiratory tract is a heterogeneous cellular ecosystem consisting of pseudostratified epithelium that contains multiciliated, mucus‐secreting goblet cells, tuft, neuroendocrine cells and a population of basal cells.^32^ Secretory and multiciliated cells perform mucociliary clearance, a self‐clearing mechanism that removes inhaled particles from the upper airways, preventing their transit to the deeper more distal areas of the lung.^33^ Low‐level mucus production by the healthy airway forms a protective layer that is important for both host defence and immune homeostasis.

In the distal lung, the respiratory bronchi branch into bronchioles then terminal bronchioles that extend into the alveolar ducts and alveolar sacs. Within the alveolus, two morphologically and functionally distinct populations of epithelial cells are found, alveolar type I (ATI) and type II (ATII) epithelial cells. Maintenance of the alveolar epithelium during homeostasis and regeneration after lung injury *in vivo* are fuelled by the surfactant‐producing ATII cells, which can renew and differentiate into ATI cells, specialized for gas exchange.^34^ A proportion of ATII cells have higher ‘clonogenic potential’, they form discrete ‘renewal foci’ often localized within the perivascular regions at the edge of the lung following injury,^35^ suggesting these specific locations within the tissue are ‘hot spots’ for alveolar renewal. It is unclear whether the proposed ATII cell heterogeneity is the result of differences in cell‐intrinsic potential or microenvironmental regulation. The anatomical location(s) and plasticity of these cells are reviewed and described in detail by Basil *et al*.^36^

**Figure 1:**
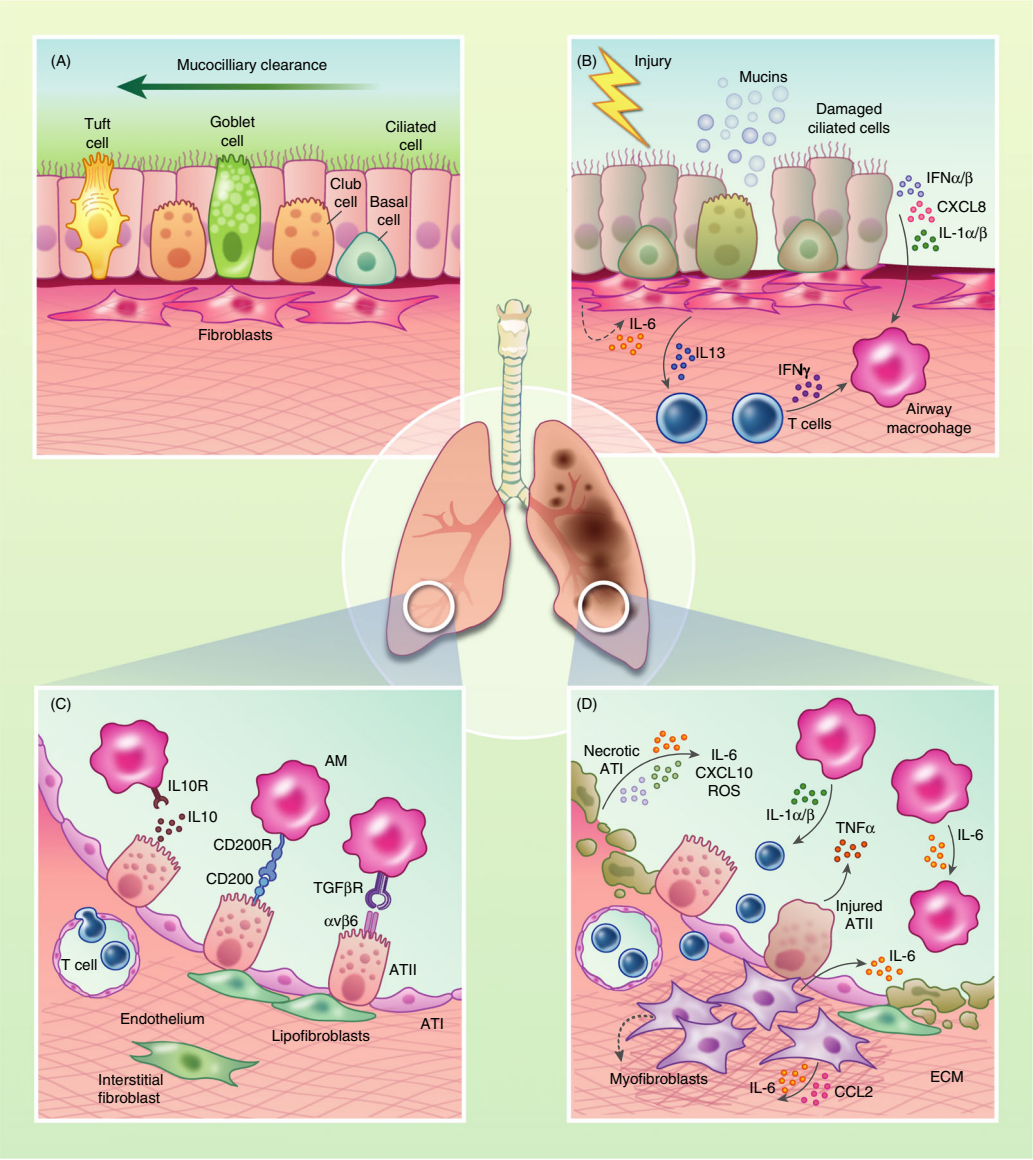
Diagram showing stromal–immune cell communication in the upper and distal airways in lung homeostasis and disease. (A) The upper airways (trachea and main stem bronchi) are lined by a pseudostratified epithelium consisting of secretory (club and goblet), ciliated, tuft and basal cells. Ciliated cells facilitate the removal of foreign particles and debris *via* mucociliary clearance (thick red arrow). A layer of stromal fibroblasts is located beneath the basement membrane. (B) Epithelial cells attempt to clear infections by inducing apoptosis or by dedifferentiation (damaged epithelium shown in red). The epithelium can secrete mucins, and a variety of cytokines and chemokines (solid arrows) that activate and attract immune cells to the lung. Secreted factors include, but are not limited to, interferon‐alpha/beta (IFN‐α/β), C‐X‐C motif chemokine ligand 8 (CXCL8) and interleukin‐1 alpha/beta (IL‐1α/β). These factors drive stromal fibroblast proliferation (dotted curly arrows) and recruit airway macrophages to sites of injury. Airway macrophages and fibroblasts release soluble factors, for example interleukin‐13 (IL‐13). Activated T cells in the lung can produce interferon‐gamma (IFN‐γ) and exert their direct effects on macrophages and stromal cells. (C) In the homeostatic lung, alveolar macrophages (AM) are resident in the alveolar space (blue area) while fibroblasts are found in the interstitium (beige area). AMs are regulated by the epithelium through their interactions with CD200, expressed by type II alveolar cells (ATII) and transforming growth factor‑β (TGF‐β) tethered to the epithelial cell surface by αvβ6 integrin, and with secreted interleukin‑10 (IL‑10). (D) In the injured alveolus, there is apoptosis or necrosis of the epithelium (AT1 and ATII), denudation of the basement membrane, influx of inflammatory cells and activation of macrophages, with release of proteases, oxidants (ROS), cytokines and other inflammatory mediators. These factors (solid arrows) include, but are not limited to, interleukin‐6 (IL‐6), C‐X‐C motif chemokine ligand 10 (CXCL10) and interleukin‐1 alpha/beta (IL‐1α/β). In the pulmonary interstitium, activated fibroblasts become ECM‐producing myofibroblasts, proliferate (dotted curly arrows) and release inflammatory chemokines IL‐6 and C‐C motif chemokine ligand 2 (CCL2).

Complex multidirectional interactions between ATII cells and fibroblasts help maintain the alveolar unit. For example, fibroblasts communicate with endothelial cells and ATII cells *via* gaps in the basement membranes and secretion of growth factors.^35^ Localization to the alveolar space is a unique feature of alveolar macrophages (AM). Their activation is tightly regulated to limit inflammatory responses by several cell–cell interactions with epithelial cells. Epithelial cells deliver inhibitory signals to AMs *via* CD200–CD200R interactions,^17^ while interleukin‐10 (IL‐10) and αvβ6‐integrin tethered transforming growth factor‐beta (TGF‐β) block pathways that lead to inflammation. Following epithelial injury/loss, numerous molecules, including IFN‐γ, IL‐1 and TNF‐α, drive AM activation. Macrophages can also communicate with ATII cells by transmitting anti‐inflammatory signals through gap junctions and secreting cytokines that promote epithelial cell proliferation.^37^ This exemplifies cellular communication across a local environment and is illustrated in Figure 1. Critically, each cell relies on healthy functioning neighbours, with damage altering the behaviour of multiple cell types.

Parenchymal lung fibroblasts are located mainly within the interstitial space, while airway fibroblasts have a predominantly subepithelial distribution. Heterogeneity of fibroblasts in the murine and human lung during homeostasis and disease is increasingly recognized^38^, ^39^, ^40^; although there is no consensus on fibroblast lineages, subtypes, biological properties or plasticity. Pulmonary fibroblast subpopulations differ in surface marker expression (e.g. presence or absence of Thy1) and have specific growth characteristics and antigen presentation functions.^41^ Alterations in cytokine production, lipid content and cytoskeletal composition have been demonstrated,^11^, ^42^, ^43^ suggesting fibroblasts may either be derived from different cell types, represent different stages of activation or are influenced by the surrounding milieu. A recent study by Xie *et al*.^40^ defined six mesenchymal cell types in the normal mouse lung: myofibroblasts, Col13a1 matrix fibroblasts, Col14a1 matrix fibroblasts, lipofibroblasts, mesenchymal progenitors, mesothelial cells and endothelial cells. Fibroblasts in different tissues may provide location‐specific signalling to neighbouring cells and positional cues for wound healing and tissue regeneration. This concept is termed ‘positional memory’.^44^

## Stromal cells display immune effector functions and have ‘poised immune potential’

Following infection, pulmonary epithelial cells and fibroblasts secrete a wide range of chemokines and cytokines that can control cell survival, influence proliferation and participate in the recruitment and activation of immune cells in the lung. Both cell types express pattern recognition receptors (PRRs), such as Toll‐like receptor (TLRs), react to stress and secrete antimicrobial peptide mediators.^45^, ^46^ Airway epithelial cells secrete anti‐inflammatory cytokines to facilitate local clearance of apoptotic cells.^47^ Fibroblasts actively modulate immune cell behaviour by adjusting either the local cellular or cytokine microenvironment. For example, fibroblasts amplify and perpetuate the immune response *via* constitutive and inducible expression of C‐C and CXC chemokines or inhibit the recruitment of circulating immune cells to sites of tissue injury.^3^ These functions were highlighted by a recent study by Krausgruber *et al*.^25^ where the authors analysed fibroblasts, epithelial cells and endothelial cells from 12 different tissues in mice in homeostasis and following a systemic viral infection with LCMV. This study identified the expression of chemokine genes by stromal cells, for example Ccl25, Ccl21a, Cxcl10, Cxcl12, Ccl2 and Ccl13, that function to attract immune cells. Stromal cells expressed genes encoding ligands and receptors including B2m, Cd74, Sdc1, Sdc4, Tnfrsf1a and Vcam1, which facilitate communication with immune cells such as B cells, macrophages and T cells.^25^

In this study, different stromal cell subtypes from the same organ displayed more transcriptional similarity among shared ‘immune genes’ than the same stromal subtypes isolated from different organs, suggesting co‐ordination of their response.^25^ Though this study was performed in mice, the alteration of cellular communication networks from baseline stromal–stromal to stromal–immune interactions in response to viral infection is consistent with findings from Vieira Braga *et al*.,^26^ in the human lung.

Interestingly, Krausgruber *et al*. suggest some immune genes expressed by stromal cells have an ‘unrealized immune potential’ and are ‘poised’ for expression (ATAC‐seq showed chromatin in an open state). ‘Poised’ genes have an open, accessible promoter but with low levels of gene expression in stromal cells. This indicates the potential of these genes to facilitate a rapid response by stromal cells when infection occurs, protecting the organ in which they reside.^25^ This was further confirmed by infection of mice with LCMV; genes with ‘unrealized potential’ were now expressed in fibroblasts and endothelial cells. However, some key response modules, including IFNGR1, display high chromatin accessibility across all samples, suggesting the rapid ability to respond to type 1 IFN is common.^25^ It should be noted that stromal cells from mucosal sites (e.g. lung and gut) had very few genes that displayed ‘unrealized immune potential’, this might be due to the fact that stromal cells at barrier sites are constantly exposed to a variety of stimuli and are the ‘first line of defence’.

Recent work from Labarta‐Bajo *et al*.^48^ found that the gut epithelial response to LCMV‐Cl 13 infection was driven by IFNAR signalling. Continuous replication of LCMV‐Cl 13 occurred in haematopoietic cells and fibroblasts, but not in the epithelial cells of the intestine. Type 1 IFN signalling, driven by infected haematopoietic cells and fibroblasts, drove increased intestinal epithelial cell (IEC) expression of T‐cell chemo‐attractants, Cxcl9 and Cxcl10, and promoted recruitment of CD8 T cells.

Further profiling of stromal cell chromatin accessibility in cells at mucosal sites is needed, particularly in infection challenge models where it is feasible to compare viral replication and clearance. Localized lung infection with respiratory viruses (e.g. influenza) represent a more organ‐specific challenge. ATAC‐seq performed prior to, during and following infection would reveal infection and immune‐driven alterations in interactions between immune and stromal cells, enabling further characterization of the phenomenon of unrealized immune potential.

## Lung stromal response to influenza virus infection

Influenza A virus (IAV) is a common respiratory pathogen causing respiratory infections that are a significant cause of morbidity and mortality worldwide.^49^, ^50^ The World Health Organisation (WHO) estimates annual epidemics result in about 3 to 5 million cases of severe illness and about 290 000 to 650 000 respiratory deaths. The lung contains various heterogeneous populations of epithelial cells that are altered following IAV infection. Lost or damaged cells can be replaced by a number of biological processes. One such process is transdifferentiation in which cells can regress to a point where they can switch lineages or can directly turn into a distinct cell type.^51^ Dedifferentiation of airway cells (e.g. basal cells, secretory cells, tuft cells) has been observed in several studies following lung injury,^52^ indicating a high degree of cellular plasticity. The differentiation of intrapulmonary basal‐like cells following influenza virus‐induced injury is reviewed extensively here.^53^ Mice and humans exhibit dramatic lung regeneration following IAV‐induced injury involving numerous populations of progenitor cells.^54^, ^55^, ^56^, ^57^ Several studies have employed lineage tracing strategies; one caveat of this approach is the possibility that transdifferentiating cells are identified because the putative originating cell marker is actually an injury‐responsive gene. To avoid inappropriate marking of cell types, lineage labelling should be performed far in advance of induced injury and investigate equivalent cell types in the steady state.

The pulmonary epithelium is immunologically active and essential for regulating immune responses in the lung.^58^ Epithelial dysplasia in mice after infection with IAV is associated with prolonged inflammation.^59^, ^60^ Several groups have also identified the presence of intermediate or transitional ATI/ATII cells in the mouse and human lung following injury.^6^, ^7^, ^8^, ^61^ These transitional cell states are found in response to influenza virus infection,^6^ in response to bleomycin‐induced pulmonary fibrosis and in recent landmark studies in the lungs of idiopathic pulmonary fibrosis (IPF) patients.^62^, ^63^ Choi *et al*.^61^ identified alterations in communication between ‘transitional’ epithelial cells and macrophages following acute lung injury. Interstitial macrophages were present in larger numbers post‐injury and were adjacent to lung ATII cells. These macrophages serve as a major producer of IL‐1β, which promoted growth of alveolar (ATII) organoids *in vitro*.^61^ Similar findings were observed by Katsura *et al*.,^64^ following IAV infection, whereby immune‐derived inflammatory cytokines, IL‐1 and TNF‐α, released from inflamed areas serve as a facultative ‘inflammation‐associated niche’, driving proliferation of ATII cells.

The function and behaviour of fibroblasts are regulated both by biochemical and by physical cues.^65^ IAV infection can cause abnormal remodelling of lung parenchyma depending on the severity of infection.^59^ The pulmonary ECM continuously provides cells with tightly choreographed spatiotemporal changes in biochemical and biophysical signals to regulate tissue‐specific cell functions and fate. Additionally, the ECM is itself a reservoir for numerous growth factors and cytokines, which are crucial for cell differentiation and proliferation.^66^ During infection, lung fibroblasts respond to damaged epithelial cells, transmit inflammatory signals and modify the ECM to generate a tissue environment promoting immune responses to infection.

A study by Boyd *et al*.^10^ identified two subsets of inflammatory fibroblasts, interferon‐responsive fibroblasts that peak around day 3 post‐infection and damage‐responsive fibroblasts that peak around day 12. At day 10 post‐infection, when anti‐IAV T‐cell responses peak, damage‐responsive fibroblasts, expressing the metalloproteinase, ADAMTS4, were found in areas of interstitial inflammation at distal airways.^67^ The presence of the ECM protease ADAMTS4 resulted in increased migration of CD8^+^ T cells across versican‐coated membranes in a Transwell assay and was associated with increased disease *in vivo*. By modifying the extracellular matrix, fibroblasts can influence the migration of immune cells that enter inflamed tissues, and, at least in this study, demonstrate immune and stromal cells collaborating to amplify immunopathology. Interestingly, ADAMTS4 is also enriched in the fibrotic human lung.^63^

**Figure 2:**
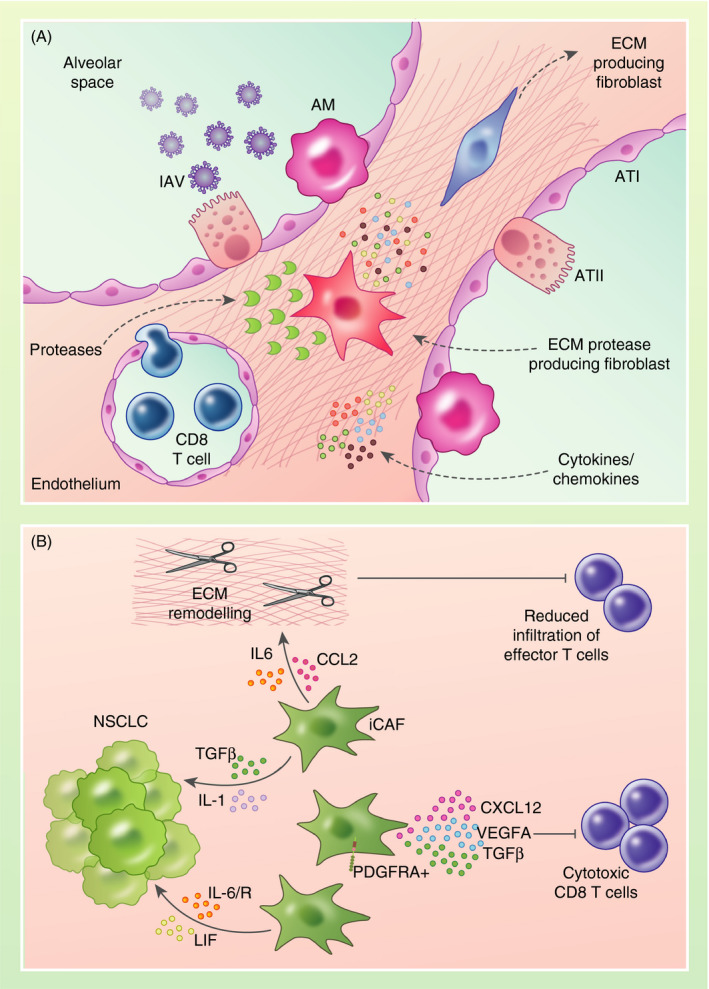
Fibroblast modification of the ECM regulates T‐cell entry into the lung in IAV infection and cancer. (A) Following IAV infection, ECM modified by fibroblast activity integrates innate immune signals to regulate the adaptive immune environment of the lung. Fibroblasts produce inflammatory chemokines and cytokines that drive ATII proliferation, for example interleukin‐1 beta (IL‐1β) and tumour necrosis factor‐alpha (TNF‐α). ECM protease‐producing fibroblasts degrade the ECM allowing T cells to migrate from the vasculature into the lung tissue to combat infection. Components are labelled with dotted line arrows. (B) Cancer‐associated fibroblasts (CAFs) also modify the ECM and regulate T‐cell entry into the tumour microenvironment. Inflammatory CAF (iCAF) subtypes secrete numerous chemokines and cytokines (indicated by curly arrows), such as transforming growth factor‐β (TGF‐β), interleukin‐6 (IL‐6), interleukin‐1 (IL‐1) and lymphocyte inhibitory factor (LIF) that promote the growth and proliferation of tumour cells. Other soluble factors released by iCAFs such as C‐X motif chemokine ligand 12 (CXCL12) and vascular endothelial growth factor A (VEGFA) inhibit (blunted arrow) the antitumour immune response *via* suppression of cytotoxic CD8 T cells. Finally, CAFs can synthesize ECM components (MMPs and collagens), and modification of the ECM by IL‐6 and C‐C motif chemokine ligand 2 (CCL2) contributes to increased ECM stiffness, which in turn reduces (blunted arrow) the infiltration of effector T cells to the tumour site. NSCLC, non‐small‐cell lung cancer.

In the absence of fibroblast‐derived ADAMTS4, fewer CD8^+^ T cells were present in the lung and versican levels were higher.^10^ This agrees with several studies demonstrating that versican can suppress cytotoxic T‐cell responses and inhibit migration.^68^, ^69^ Cancer‐associated fibroblasts (CAFs) can perform a similar function.^5^ Immunomodulatory CAFs found in breast cancer can regulate the migration of T cells and limit their cytotoxic functions; notably, patients with low levels of these CAF cells had a survival advantage.^70^ Figure 2 illustrates how fibroblast modification of the ECM regulates T‐cell migration.

The ageing process can impact the lung microenvironment. A recent study by Goplen *et al*.^71^ showed the aged mouse lung displays defective CD8^+^ T‐cell immunity and increased tissue damage following IAV. Excessive accumulation of tissue‐resident influenza‐specific, memory CD8 T cells (TRM) occurred in the parenchyma of the aged lung. CD8 TRM cells require TGF‐β during development and to persist. In aged lungs, elevated TGF‐β levels were accompanied histologically by chronic inflammation and by fibrotic sequelae after viral pneumonia.^71^ A possible explanation for these findings is that a net gain in lung TGF‐β levels, as seen with ageing, may act on lung parenchymal fibroblasts, to promote the production of collagen and amplify the fibrotic phenotype.

## Specialized microenvironments in the infected lung: friends or foes?

Lung macrophage infection by the bacteria *Mycobacterium tuberculosis* (*M.tb*) drives a localized inflammatory response, inducing chemokine secretion, and promotes recruitment of macrophages, neutrophils and T cells.^72^ These cells form an organized structure called a granuloma. Later, T and B cells surround the granuloma and are further enclosed by fibroblasts, demarcating the peripheral structure and perhaps containing the infection.^73^ In the *M.tb‐*infected lung, alveolar macrophages, bronchial epithelial cells and fibroblasts upregulate expression of ECM‐degrading matrix metalloproteinases (MMPs).^74^, ^75^ Degradation of the ECM can destabilize the granuloma. In the human lung, airway epithelial cells and fibroblasts adjacent to granulomas produce MMP3 in response to lymphocyte‐derived IL‐17.^76^ Expression of myeloid‐derived oncostatin M (OSM) in human granulomas promotes the production of MMP1 and MMP3 by lung fibroblasts.^77^ These data indicate that stromal MMP production promotes tissue destruction, amplifies the inflammatory response and facilitates the dissemination of *M.tb* throughout the lung parenchyma.

Pulmonary viral infection is associated with the formation of ectopic‐inducible bronchus‐associated tissue (iBALT) in the lung.^60^, ^78^ iBALTs are located in the perivascular space surrounding large blood vessels and airways.^79^ They are an excellent example of a specialized microenvironment whereby communication between stromal and immune cells promotes immunity. Sites of iBALT aid robust B‐ and T‐cell responses to influenza virus and are highly organized, with distinct B‐cell follicles and T‐cell areas, supporting the proliferation of these cells.^80^ It is important to note that organized iBALTs with distinct T/B zones are only found in the IAV‐infected lungs of young mice and not adult mice, as such, these findings require cautious interpretation.^80^, ^81^

Highly specialized stromal cells facilitate organization, maintenance and survival of leucocytes within iBALT. CD31^+^ PNAd^+^ high endothelial venules (HEV) form near the outer edges of the B‐cell follicle and serve as entry points for recirculating lymphocytes.^82^ Lymphatic endothelial cells (LECs) are found in areas surrounding iBALT and support T‐cell recruitment and survival *via* secretion of chemokines (CCL21 and CCL19). In secondary lymphoid organs, maintenance of the B‐cell follicle depends on secretion of CXCL13 by CD35^+^ follicular dendritic cells (FDCs).^83^ Podoplanin (PDPN) is a signature molecule of immunomodulatory fibroblasts.^84^ Interestingly, a non‐classical B‐cell follicle has also been described in iBALT, lacking FDCs, instead using podoplanin (PDPN)+CD35^−^ CD31^−^ CXCL12^+^ fibroblast‐like stromal cells to maintain the B‐cell area.^85^ Similar findings were observed by Denton *et al*.,^86^ whereby the production of CXCL13 by lung fibroblasts induced after IAV infection drives CXCR5‐dependent recruitment of B cells. These PDGRFα^+^ lung fibroblasts express high levels of podoplanin. Intriguingly, these CXCL13^+^ fibroblasts are functionally distinct from both FDCs and other populations of resident lung fibroblasts, indicating anatomical location within the lung is an important driver of immunomodulatory function.

Transcriptionally altered lung epithelial cells are detected following IAV infection for prolonged periods of time; some of these cell types appear to be long‐lived and express high levels of immune genes.^87^, ^88^ Heaton *et al*. characterized a long‐lived population of club cells, showing increased interferon stimulation that displayed high levels of pro‐inflammatory chemokines, Cxcl10, Ccl20 and Ccl5, after viral clearance.^87^ This is a model in which infected, surviving club cells establish a pro‐inflammatory environment at the bronchi, not only promoting increased control of new viral infections but also contributing to lung pathology. Fiege *et al*.^88^ demonstrate that in addition to club cells, ciliated epithelial cells, ATI and ATII cells can survive IAV infection. These surviving cells undergo enhanced proliferation compared with uninfected cells following IAV clearance and upregulate PD‐L1 expression. These cells evade CD8 T‐cell‐mediated killing by rapidly clearing viral infection, thus limiting immunopathology. Rane *et al*.^89^ demonstrate the appearance of solitary chemosensory cells (SCCs), a type of tuft cell, in the distal lung following IAV‐induced lung injury. These keratin‐5 (Krt5+) cells are located in close proximity to dysplastic epithelium and close to inflammatory cells, suggesting crosstalk between the epithelial and immune compartments. This may promote inflammation that drives and/or maintains epithelial dysplasia. However, these Krt5+ cells rarely resolve into ATI or ATII cells and instead form dysplastic ‘epithelial scars’ that persist through the life span of mice.^55^, ^56^ As they do not contribute to lung function, it is possible they represent areas of localized fibrosis. Replacing pulmonary alveoli with scar tissue has long‐term functional and physiological consequences for the lung.^59^, ^90^, ^91^, ^92^

## Pulmonary fibrosis promotes stromal–immune cell interactions

Lung remodelling eventually leads to resolution of injury and repair. If this fails or the process becomes self‐sustaining, uncontrolled tissue fibrosis can occur. Progressive tissue fibrosis leads to disruption of cellular architecture, loss of organ function and eventually death. Idiopathic pulmonary fibrosis is a chronic progressive form of interstitial lung disease, characterized pathologically by heterogeneous areas of inflammation and fibrosis in the lung.^93^ A prevailing theory in IPF, broadly applicable to mouse models of lung fibrosis, is that the alveolar epithelial cell is the initial site of insult/injury. Mechanisms of pulmonary fibrosis are reviewed extensively here.^94^

In the bleomycin model of pulmonary fibrosis, Strunz *et al*.^7^ identified a unique transitional cell state (Krt8+ alveolar differentiation intermediate state (ADI)) with a unique transcriptional signature. This included a p53‐driven gene programme and features of cellular senescence, preceding the regeneration of ATI cells. These findings are supported by Kobayashi *et al*.,^8^ who identified alveolar epithelial cells that undergo exhaustive stretching during transdifferentiation that makes them vulnerable to DNA damage and display enrichment of binding sites for transcription factors, including TP53, ETS1, NF1, ATF3 and SOX4. Strunz *et al*.^7^ demonstrated ADI expression of profibrogenic factors and a distinct connectome of receptor–ligand pairs, between endothelial cells, fibroblasts and macrophages in infected compared with naïve mice. These Krt8+ ADI cells form a unique cellular niche during the fibrogenic phase of tissue repair, concurrent with myofibroblasts and M2 macrophages, displaying differences in communication with these cell types and the alveolar epithelium.

Reyfman *et al*.^95^ identified profibrotic macrophages in lungs of humans and mice with pulmonary fibrosis. The existence of these profibrotic macrophages was validated by recent landmark studies in the IPF lung.^62^, ^63^

Adams *et al*.^62^ showed that profibrotic macrophages were elevated in the IPF lung; these cells express SPP1, and as disease progresses, ECM remodelling genes are elevated (SPARC, GPC4, PALLD, CTSK and MMP9). During late stage disease, these profibrotic macrophages start expressing colony‐stimulating factor (CSF1), indicating a possible autocrine feedback loop for recruitment and activation. Macrophages usually provide tropic factors to fibroblasts in return for survival cues; typically, fibroblasts produce CSF1; and the CSF receptor (CSFR) is exclusively expressed by macrophages. Zhou *et al*.,^96^ identified CSF1‐CSF1R as the minimal interaction necessary to sustain fibroblast/macrophage circuitry *in vivo*. In this study, the presence of high levels of CSF1 promoted macrophage growth. A study by Joshi *et al*.^97^ identified M‐CSF/M‐CSFR signalling in monocyte‐derived alveolar macrophages as a critical regulator of the fibrotic niche. These macrophages were specifically localized to fibrotic regions in the proximity of lung fibroblasts and expressed molecules known to drive fibroblast proliferation, such as PDGFA. This suggests additional signalling increases the fibrotic milieu.

Transcriptionally distinct fibroblast subtypes have been identified, in distinct regions of the fibrotic lung. Myofibroblasts are found in subepithelial regions around airways and areas of cystic remodelling, while HAS1^hi^ (hyaluronan synthase 1) fibroblasts are restricted to the immediate subpleural region.^63^ In contrast, PLIN2+ (perilipin‐2) and other LUM+ (lumican) fibroblasts are found diffusely in parenchymal regions. Consideration of the spatial location of these altered cells within the lung confirms the importance of matching transcriptional signatures with discrete anatomical locations. However, it is not possible to determine conclusively whether the fibroblast subsets identified here represent either phenotypically distinct populations or a continuum of activation states.

Cellular interactions within IPF lungs were investigated through analysis of receptor–ligand pairs. This revealed that dominant interactions were between aberrant basaloid cells, fibroblasts, myofibroblasts and T cells.^62^ The functional capabilities of these cells and their distinct contribution to the lung microenvironment require further investigation. It is important to consider that in these studies,^62^, ^63^ the healthy/control human lung has been rejected for transplant and may exhibit inflammation. Additionally, these ‘control’ lungs are likely to have experienced acute injury, infection and/or previous environmental exposures.

## The COVID‐19 cytokine storm: do myeloid‐derived cytokines drive the activation of stromal cells?

The mechanism by which severe acute respiratory syndrome coronavirus 2 (SARS‐CoV‐2) causes lung damage has yet to be fully elucidated. SARS‐CoV‐2 infection does closely resemble that of SARS‐CoV infection, with aggressive inflammatory responses strongly implicated in causing damage to the airways.^98^ Disease severity in patients is likely a consequence of a combination of viral infection and host response; the pathophysiology of COVID‐19 is reviewed here.^99^ Importantly, interactions between immune and epithelial cells correlate with disease severity.^100^ Enhanced plasma concentrations of TNFSF14, EN‐RAGE and OSM correlate with disease severity; the receptors for these myeloid‐derived cytokines are highly expressed by human lung fibroblasts and are implicated in fibrotic remodelling of the lung.^101^ It is plausible that these myeloid‐derived cytokines activate the lung stromal microenvironment. Taken together, these findings indicate stromal–immune interactions are likely more pronounced in severe COVID‐19 patients and may ultimately lead to end‐stage organ damage.

Understanding the long‐term consequences for the virally infected lung is of critical importance and could underlie the distinct severity of responses observed in COVID‐19 patients. We need to ascertain therefore whether past infections have consequences for future immune responses *via* changes to the stromal cells. Altered stromal cells may provide immune protection directly, for example *via* antiviral cytokine or generate protective immunity through communication with CD4 and/or CD8 T cells or B cells. Conversely, the expression of ‘immune genes’ by stromal cells may promote inflammation causing tissue destruction. More in‐depth and temporal analysis of stromal–immune crosstalk is required to reveal the mechanisms that underlie these protective or pathogenic interactions.

## Conclusions

Cellular communication between immune and stromal cells in the lung regulates a diverse range of biological process. Stromal cells can perform functions classically attributed to immune cells, providing immunomodulatory functions and educating local immune cells within the lung. Inflammatory fibroblasts can integrate danger signals from epithelial cells and resident immune cells to produce diverse inflammatory cytokines, ECM components and degradative enzymes that modify the local tissue environment. This illustrates that communication between immune and stromal cells is bidirectional, providing mutual support for the persistence of both cell types. Regenerative cues from both the stromal and immune compartments regulate epithelial repair spanning vast areas of the lung, and immune cell recruitment to sites of injury is tightly regulated *via* local modulation of ECM. Further elucidation of these context‐dependent interactions may provide us with novel strategies to limit excessive inflammation and maintain the normal architecture of the lung following tissue injury.

## Author contributions

J.C.W and M.K.L.M conceptualized the study with equal contribution. J.C.W drafted the manuscript and figures. M.K.L.M revised manuscript and figures. J.C.W and M.K.L.M approved the final version for submission.

## Conflict of interest

The authors have declared that no conflict of interest exists.

## Financial support

This work was supported by the Wellcome Trust [210703/Z/18/Z].

## Data Availability

Data sharing is not applicable to this article as no data sets were generated or analysed during the current study.
